# Science Shops as key intermediary structures to respond to the current health research agenda bias: Evidence from the InSPIRES project

**DOI:** 10.1111/hex.14052

**Published:** 2024-04-23

**Authors:** Aina Estany, Fredrik Niclas Piro, Jacqueline E. W. Broerse, Rosina Malagrida

**Affiliations:** ^1^ Living Lab for Health IrsiCaixa Research Institute, IGTP Badalona Spain; ^2^ Nordic Institute for Studies in Innovation, Education and Research (NIFU) Oslo Norway; ^3^ Athena Institute Vrije Universiteit Amsterdam Amsterdam The Netherlands

**Keywords:** health, participation, research agenda, research bias, research priorities, Science Shop

## Abstract

**Introduction:**

To increase the likelihood of research responding to societal needs, intermediary structures such as Science Shops are being created. Science Shops respond to research needs identified and prioritized through participatory processes involving civil society. However, these are not mainstream structures, and most research needs addressed by the scientific community are not defined by a diversity of stakeholders (including citizens) but are mostly prioritized by researchers and funders. Literature shows this often leads to bias between the research topics investigated and the research needs of other relevant stakeholders. This study analyses how 14 Science Shops contribute to decreasing bias in health research agenda setting.

**Methodology:**

We compare the research priorities identified through participatory processes by the Science Shops, which participated in the European Union‐funded project InSPIRES (2017–2021), to the available research addressed in the literature (identified in Web of Science), which we use as a proxy for current research priorities.

**Results:**

Science Shop projects contributed to decreasing the existing bias in health research agenda setting: (1) between drug and nondrug treatments and (2) between clinical trials of treatments for illnesses affecting high‐income versus middle‐ and low‐income countries, which leads to a lack of local strategies for high disease burdens in nonhigh‐income regions.

**Conclusion:**

This study provides the first evidence of Science Shops' effectiveness in addressing current biases in health research agenda setting. We conclude they could play a key role in shaping local, national and international research policies.

## 1 INTRODUCTION

To increase the likelihood that research will have an impact on society, intermediary structures are being created. One such structure is the *Science Shop*, which provides ‘independent, participatory research support in response to concerns experienced by civil society’.^1^, ^2^ Science Shops respond to research needs identified and prioritized through participatory processes involving civil society.

Science Shops emerged in The Netherlands in the 1970s, facilitated by a favourable political context that allowed university staff and students to start partnerships with civil society organizations (CSOs). Students, supervised by senior researchers, undertook research projects for free upon CSOs' requests.^3^, ^4^ In the 1980s, the Dutch Science Shop movement inspired other European countries. The establishment of those Science Shops was enhanced by the environmental movement, which motivated collaborations with emerging university departments in environmental sciences. The Science Shops concept gained further traction in the 1990s, driven by the Information and Communication Technologies revolution, which promoted a shift towards a knowledge‐based economy with emerging debates about the active role that society should have in science, moving away from the industrial economy in which society was just a receiver of scientific knowledge. The European Commission (EC) funded several projects on Science Shops at this time. In the late 1990s, the movement spread in eastern and central Europe^3^ and outside Europe to countries such as South Africa.^5^

Over the past decade, renewed attention to Science Shops has emerged with innovative approaches being used around the world. These efforts have been supported by international organizations such as the EC, which has funded new research and innovation (R&I) projects to*** promote more open and inclusive approaches influenced by different science policies. Some of these policies promoted the participation of diverse stakeholders in different phases of R&I processes, starting with collectively defining research needs.^6^, ^7^, ^8^, ^9^ Science Shops differ in the activities they conduct, how they are organized, in which context and fields they work, and how they operate.^4^ However, although no dominant organizational structure defines a ‘Science Shop’, they all aim to respond to research topics identified and prioritized with a bottom‐up approach.^1^ Despite the growing interest, their role is currently relatively small as few higher education institutions have a (well‐functioning) Science Shop.

 

Study and Conference on Improving Public Access to Science through Science Shops, Improving Science Shop Networking, Training and Mentoring of Science Shops, Public Engagement with Research and Research Engagement with Society, Enhancing Responsible Research and Innovation through Curricula in Higher Education, Enhancing the Responsible and Sustainable Expansion of the Science Shops Ecosystem in Europe),  Ingenious Science shops to promote Participatory Innovation, Research and Equity in Science

Although participatory methods are increasingly used in research, most research today still addresses topics identified and prioritized by researchers and funders (public and private) that are not necessarily aligned with the research needs of a broader community of stakeholders (including citizens).^10^, ^11^ This may result in what Knottnerus and Tugwell^12^ describe as a research agenda bias in clinical research. They point out that this bias is reflected in the ‘striking disbalance’ between efforts in: (1) research fields within drug research, such as research on the effectiveness of starting or prescribing drugs versus research on drug cessation (withdrawal, deprescribing, stopping, discontinuation, or reduction of drug treatment), diagnosis and prognosis; (2) research fields in pharmaceutical versus nonpharmaceutical interventions, like psychotherapy, health education or rehabilitation which ‘are severely underserved’; (3) proportion of research and burden of diseases, both within high‐income countries and between high and nonhigh‐income regions, such as common infectious diseases and neonatal disorders in South Asia and (4) drug research versus research on related healthcare policies such as the availability of essential medicines in low‐income countries.^12^

A bias in health research is also described by Crowe et al.,^10^ who compared the research topics addressed in registered trials with the research needs identified and prioritized by patients and clinicians over the same time period (2003–2012). The main priorities addressed in the internationally registered clinical trials during that time focused on drug evaluations. However, the priorities identified by patients and clinicians emphasized the importance of nondrug interventions, such as education and training, psychological therapy, or social care. Tallon et al.^11^ in their exploration of research priorities on the management of osteoarthritis of the knee, found a similar bias towards the development of new drugs. When patients and clinicians were asked to identify their priorities, they wanted more rigorous evaluation of the effects of physiotherapy, surgery, education, and coping strategies rather than more studies of drugs.

If we do not address the current research agenda bias, which is leading to key unmet needs regarding nondrug treatment research, we will not be able to achieve integrated care. Since integrated care seeks to better co‐ordinate care around people's needs, it is important to also align research agendas with those needs. One solution to address the research agenda bias and to respond to ‘the much too often hidden, implicit international and national research agendas’^12^ is to involve different types of stakeholders in the research agenda‐setting process.

Science Shops are one of the intermediary structures responding to research needs identified and prioritized through the participation of stakeholders.^2^, ^3^, ^9^, ^13^ Structures that follow more open and inclusive approaches use different multistakeholder participatory methods to set research priorities after identifying the challenge. They follow two different approaches to workflow: (1) one‐off approaches, facilitated with participating stakeholders having fixed roles, including methods such as interviews, surveys, workshops and science cafés, often combining two of these methods, and (2) iterative approaches, characterized by action‐learning spirals with collective reflexive and learning processes that bridge roles and positions of multiple‐stakeholders while facilitating knowledge integration for joint decision‐making, such as the Dialogue Model, the System‐Oriented Dialogue Model, the Delphi Method or the methods used by the James Lind Alliance.^9^, ^14^, ^15^, ^16^, ^17^, ^18^, ^19^ They also have different approaches for identifying, recruiting, retaining and ensuring inclusion of diverse and representative stakeholders with multiple interests and concerns in safe and cooperative arrangements and for integrating (transdisciplinary) knowledge and dealing with the complexity of challenges.^18^, ^20^ Once research priorities are defined, they reformulate the research question and design and implement participatory research projects with methods such as citizen science or community‐based participatory research^21^ ‘while also continuing to rely on approaches wherein problems are solved by practice of combining and adapting existing knowledge from different sources without the “scientific research” dominating the process’.^9^

However, little is known about the effectiveness of these approaches in addressing research agenda bias.

Our study analyses whether Science Shops contribute to decreasing bias in health research agenda setting. We compared the research priorities identified through participatory processes by 14 Science Shops that participated in the European Union (EU)‐funded project InSPIRES and the available research addressed in the scientific literature indexed in the Web of Science (WoS), which we use as a proxy for current research priorities.

## 2 METHODOLOGY

The research was conducted within the framework of the EU‐funded project InSPIRES (Ingenious Science Shops to promote Participatory Innovation, Research and Equity in Science, https://www.inspiresproject.com/) which ran from 2017 until 2021. The project aimed to bring together different stakeholders across and beyond Europe to co‐design, jointly pilot, implement and roll out innovative models of Science Shops with participatory approaches. This study analyses data from projects conducted by the 14 Science Shops that participated in the InSPIRES project and from publications in WoS during the same period conducted worldwide and in specific countries where the Science Shops were located and/or performed their research.

The Science Shops were located in Benin, Bolivia, Ecuador, France, Greece, Hungary, Italy, The Netherlands, Romania, Spain, Tunisia, Turkey and Uganda (13 countries and 14 Science Shops with 2 Science Shops located in Spain), and some of them performed their research in other countries, such as Nepal and Ecuador (where no local Science Shops were involved). The Science Shops were committed to identifying and prioritizing research topics through participatory processes, as described in the framework for Science Shop processes developed within InSPIRES.^9^ Eight of the Science Shops were InSPIRES partners and they were located in: Europe (Spain, The Netherlands, France, Hungary, Italy), Africa (Tunisia) and South America (Bolivia). The other six were associated partners recruited through an open call, and were located in Europe (Greece, Romania, Turkey), Africa (Benin, Uganda) and South America (Ecuador). The next sections describe the methods for the comparative analysis of Science Shops projects with the publications indexed in WoS, applied within two different phases: (1) data collection and selection of Science Shop research projects and (2) analysis of research fields.

### 2.1 Phase 1: Data collection and selection of science shop research projects

The data set included basic descriptive information from each Science Shop project. Table 1 shows the categories of data collected for each project: (1) basic descriptive information, (2) characteristics of the process for identifying and prioritizing research needs (e.g., number and diversity of stakeholders involved, participatory methods applied, level of participation achieved), (3) topic (e.g., human immunodeficiency virus [HIV], Chagas, Leprosy), (4) subtopics addressed (e.g., HIV treatment, HIV‐related stigma) and (5) research field as defined by NordForsk, a funding agency under the Nordic Council of Ministers, which produced bibliometric analyses of the Nordic countries on a regular basis during the period 2009–2019^15^ (e.g., Social sciences, natural sciences, humanities, engineering). Each Science Shop provided this data in a shared file delineating the data categories and a description of each of the categories. The level of participation was categorized according to Arnstein's Ladder of Citizen Participation^16^ (Box 1) which includes participatory and nonparticipatory levels that were useful to be able to exclude the projects that applied nonparticipatory methods. Bilateral online meetings were held with Science Shop representatives to ensure that the instructions for collecting the data were clear.

**Table 1:** Data collected for each Science Shop research project conducted within the InSPIRES period (2017–2021).

Categories of data collected for each Science Shop research project		
1	Basic descriptive information	
	1.1 Name of the Science Shop research project	
	1.2 Research aim	
	1.3 InSPIRES partner who led the research	
	1.4 Country where the partner is located	
	1.5 Country where the project was implemented	
	1.6 Period of implementation	
2	Characteristics of the process to identify research needs	
	2.1. Participatory methods	Description of approaches and methods for engaging the different stakeholders in identifying the research needs.
	2.2. Number and diversity of stakeholders involved	Because the degree of different stakeholders' involvement varies according to the type of social demand,^9^ we collected data on the diversity of stakeholders involved in the identification of research needs (e.g., researchers, policy makers, education community or business and industry representatives).
	2.3. Level of stakeholder participation	Description of the level of participation achieved during the process to define research needs. The level was described according to Arnstein's Ladder of Citizen Participation,^16^ which ranges from manipulating the community to support a chosen research need to citizen control, where the community decides the priority research need.
3	Broad topic and topic	Broad topics included ‘Health’, ‘Environment’, ‘Health and Environment’ and ‘Other’. Topics could include, for example, HIV/AIDS, hepatitis, mental health or physical activity.
4	Subtopic of a topic	Information about the topic that more specifically defines the research's main focus (e.g., for a project tackling the topic of HIV, the research subtopic could be ‘HIV‐related stigma’).
5	Research field	Information about research fields was extracted from the Web of Science database, which has 259 subject categories used for classifying journals (not individual papers). All journals are classified within one or more of these categories. NordForsk^22^ proposed a reclassification of these fields into 16 broad categories, such as ‘Biomedicine and molecular biosciences’ (including subject categories such as virology, immunology, and pharmacology and pharmacy) or ‘Social sciences’ (including subject categories such as sociology, education and communication) (see Supporting Information S1: Appendix A). NordForsk's reclassification was based on a network analysis approach: that is, how journals' research fields correspond to publications from journals that cite each other. Because the NordForsk analysis is based on journals and not topics, it distinguishes between different types of research related to the same disease. This means, for example, that papers on HIV may be differently classified. A paper on HIV from *Health Policy & Services* will be classified as ‘Health sciences’, while an HIV‐related paper in *Pharmacology and Pharmacy* will be classified as ‘Biomedicine and molecular biosciences’.

Abbreviations: AIDS, acquired immunodeficiency syndrome; HIV, human immunodeficiency virus.

BOX 1 **Levels of participation according to Arnstein's Ladder of Citizen Participation**

Levels of citizen power with decreasing degrees of decision‐making:

8—Citizen control citizens obtain full managerial power.

7—Delegated power powerholders hand over some degree of decision‐making power to citizens.

6—Partnership citizens can negotiate and engage in trade‐offs with traditional powerholders, ultimately sharing decision‐making responsibilities.

Levels of tokenism:

5—Placation citizens are allowed to advise by having a (few) seat(s) on committees or boards, but they are easily outvoted by powerholders or powerholders retain the right to decide whether or not to follow their advice.

4—Consultation citizens are allowed to be heard through surveys, interviews, neighbourhood meetings, and public hearings, but they lack the power to ensure that their views will be heeded by the powerful.

3—Informing citizens are informed but no mechanisms are put in place for feedback— one‐way flow of information.

Levels of nonparticipation:

2—Therapy powerholders set up public participation to convince citizens that they are the problem and they need to be ‘cured’.

1—Manipulationpowerholders invite the public to participate with the express purpose of ‘educating’ them—participation as a public relations tool.

Source^23^

To demonstrate that Science Shop research projects addressed research needs identified and prioritized through participatory processes, characteristics of the process to identify research needs (Category 2 in Table 1) were analyzed. A descriptive analysis was conducted defining the percentages of types of participatory methods used in the Science Shops, the diversity of stakeholders involved during the identification phase, and the level of stakeholder (non‐)participation (cf. Arnstein's Ladder of Participation).

**Figure 1:**
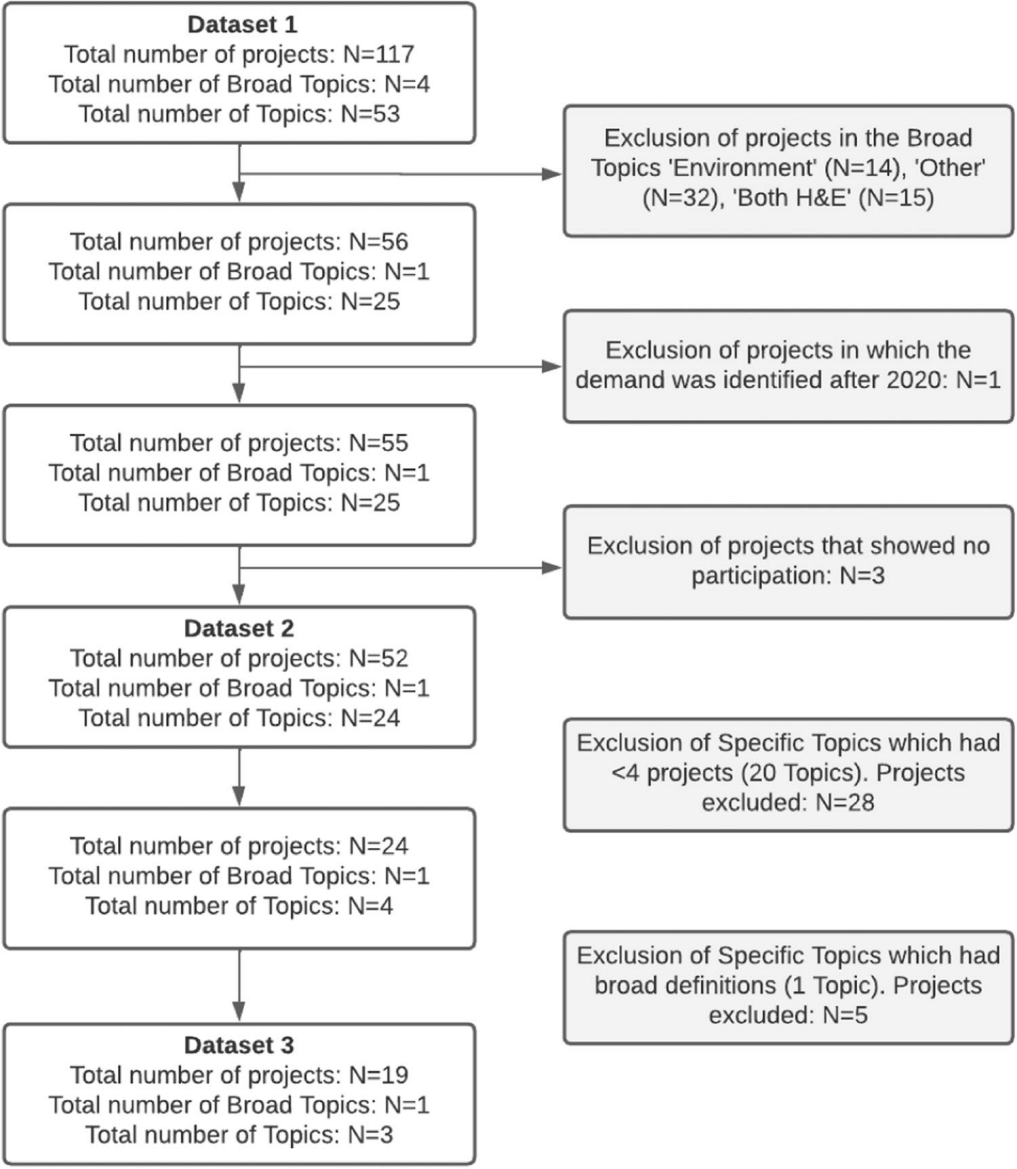
Flowchart of selected and discarded projects and topics leading from Data Sets 1 to Data Sets 2 and 3.

Finally, three data sets were defined with different inclusion criteria, as described in Table 2 and Figure 1.

**Table 2:** Description of each data set and the inclusion and exclusion criteria used.

Data set	Inclusion and exclusion criteria	Data
Data Set 1: InSPIRES Science Shop research projects (total)	–	117 projects on 4 broad topics (Health, Environment, Health and Environment and Other) and 53 topics
Data Set 2: Selected InSPIRES Science Shop research projects	(1) Projects focusing on the broad topic Health. Projects classified with Environment, Health and Environment and Other were excluded. (2) Projects performed within January 2017 to December 2020 (those conducted in 2021 were not included as they were not completed when the analysis was made). (3) Projects in which the topic had been identified and prioritized with methods that were labelled as nonparticipation, based on Arnstein's Ladder of Participation, were excluded.	52 projects on 24 topics were included in Data Set 2, and 65 projects were excluded
Data Set 3: Topics	(1) Topics with broad definitions were excluded due to possible bibliometric analysis difficulties as they cover issues that may be conceptually completely independent of each other. For example, ‘mental health’ is a broad topic, as it includes different pathologies such as anxiety, bipolar disorder, schizophrenia, depression and eating disorders. ‘Stakeholder engagement in health processes’ is also a broad topic as it includes different concepts such as participatory research agenda setting, patient engagement, citizen science or community‐based participatory research. (2) After excluding projects focused on broad topics, we excluded all topics covered by less than two projects, as they were less representative of the overall work that took place within the InSPIRES projects.	19 projects on 3 topics included in Data Set 3, and 33 projects on 21 topics excluded (Figure 1). The final three topics were HIV/AIDS (10 projects: 4 in Spain, 2 in Bolivia, 1 in Tunisia, 2 in The Netherlands, 1 in collaboration between Bolivia and Ecuador), Chagas (five projects, four in Bolivia, one in Spain) and Leprosy (four projects in The Netherlands). Therefore, the data set includes different numbers of projects implemented by each Science Shop. Supporting Information S1: Appendix B shows the final selected projects for each topic.

Abbreviations: AIDS, acquired immunodeficiency syndrome; HIV, human immunodeficiency virus.

### 2.2 Phase 2: Analysis of research fields

In this phase, we conducted a bibliometric analysis comparing the research fields in the selected projects in Data Sets 2 and 3 with research fields in the publications in the WoS database. WoS is the world's largest and most authoritative database for studying publication output,^24^ covering 34,000 journals across all fields of science, although its coverage of Arts, Humanities and Social sciences is less complete as its coverage of Science, Technology, Engineering and Mathematics and medical fields. Similarly, the database has an overrepresentation of English‐language journals,^25^, ^26^ and thus an underrepresentation of, for example, Spanish‐language journals,^27^ although in recent years, many South and Latin American journals have been indexed in WoS. Despite these shortcomings, it is still the most used database for studying publication output in the Social sciences as well as other journal‐oriented fields, as very few countries have national databases with complete coverage of all Social sciences output.

First, we identified all research publications related to the topics HIV/AIDS, Chagas and Leprosy in 2017–2020. We searched WoS article titles, abstracts and keywords using the search terms specified in Table 3. The search terms were considered unique for each specific disease: for example, when mapping papers about Chagas, the search terms used included ‘Chagas’ and the drug names ‘benznidazole’ and ‘nifurtimox’, the main treatments for this disease. These search terms were defined with the support of two clinical specialists. For subtopics, we used the same keywords as for the topics, adding the condition to also look for keywords related to the specific subtopic (e.g., ‘stigma’ and ‘discrimination’ in the case of HIV‐related stigma).

**Table 3:** Search terms used to analyse the representation of topics and subtopics in Web of Science.

Topic and Subtopic		Search terms
Topic	HIV/AIDS	HIV, AIDS, Antiretroviral therapy, Nucleoside analogue reverse transcriptase inhibitors, Tenofovir alafenamide, Emtricitabine, Emtriva, Vemlidy, Lamivudine, Abacavir, Ziagen, Integrase inhibitors, Bictegravir, Dolutegravir, Tivicay, Raltegravir, Isentress
Subtopic	HIV‐related stigma	(Stigma, Discrimination) & the search terms above
Topic	Chagas	Chagas, American ripanosomiasis, Benznidazole, Nifurtimox, Trypanosoma cruzi
Subtopic	Diet and Chagas patients	(Diet, Nutrition) & the search terms above
Topic	Leprosy	Leprosy, Hansen's disease, Dapsone, Diaminodiphenyl sulphone, Rifampicin, Rifampin, Clofazimine, Lamprene, *Mycobacterium leprae*, *Mycobacterium lepromatosis*
Subtopic	Leprosy Postexposure prophylaxis	(Postexposure prophylaxis, PEP) & the search terms above

Abbreviations: AIDS, acquired immunodeficiency syndrome; HIV, human immunodeficiency virus.

Second, we identified all research publications related to ‘Health’: the search terms used were ‘Health’, ‘Medicine’ and ‘Medical’. This analysis was only carried out at the global level. However, for the topics, it was also carried out for the countries where the selected projects were implemented and for the countries where the organization coordinating the project was located: *HIV/AIDS* in Spain, The Netherlands, Tunisia, Bolivia, Nigeria and Ecuador; *Chagas* in Spain and Bolivia; *Leprosy* in The Netherlands, Nepal and Ethiopia; *HIV/AIDS‐related stigma* in Spain, Bolivia and Ecuador.

This allowed us to compare the proportion of publications at global and country levels to identify whether the Science Shops were contributing to decreasing existing bias in research fields globally, in the countries where the research was being implemented, or even in the countries where the Science Shops were located.

The second step of the mapping included classifying WoS publications and Science Shop research projects from Data Sets 1 and 2 according to NordForsk's 16 categories of research fields. This was conducted in two parts (1) broad topic: Health, and (2) topics: HIV/AIDS, Chagas and Leprosy. Next, we analyzed the proportion of publications and projects addressing each research field for the broad topic ‘Health’ and for each topic. As in the bibliometric analysis, the countries of project coordination and implementation were also considered.

The last step involved comparing the total publications and percentages of the different research fields in WoS publications and Science Shop research projects.

## 3 RESULTS

### 3.1 This section reports the results of each phase

#### 3.1.1 Science Shop research projects

**Figure 2:**
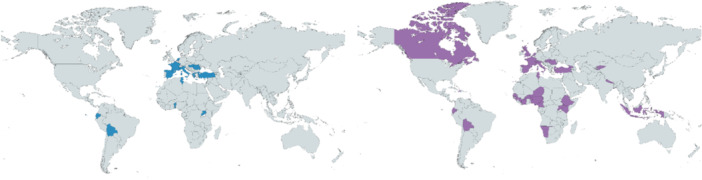
Maps displaying the countries where the Science Shops were located (left) and where research was performed (right).

We identified 117 projects implemented by 14 Science Shops located in 13 countries during the InSPIRES project lifespan (2017–2021). The projects were implemented in 30 countries (Figure 2).

**Figure 3:**
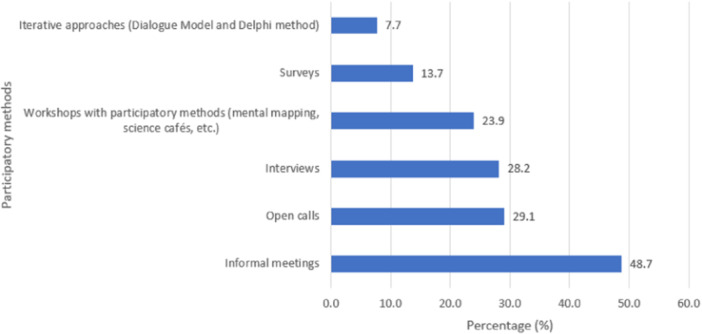
Methods used to identify and prioritize research topics and specific topics (%).

In most of these projects, the research (sub‐)topics were identified through participatory methods that ensured stakeholders' active participation in their identification and prioritization. The participatory methods used were highly varied. Most were conversations/meetings (48.7%), followed by interviews (28.2%), workshops with participatory methods such as mental mapping or Science Cafés (23.9%), open calls (19.1%) and questionnaires/surveys (13.7%) (Figure 3). In 4.3% of the projects, these participatory methods were combined with nonparticipatory methods, such as observations, literature reviews and analysis of publications in the media.

**Figure 4:**
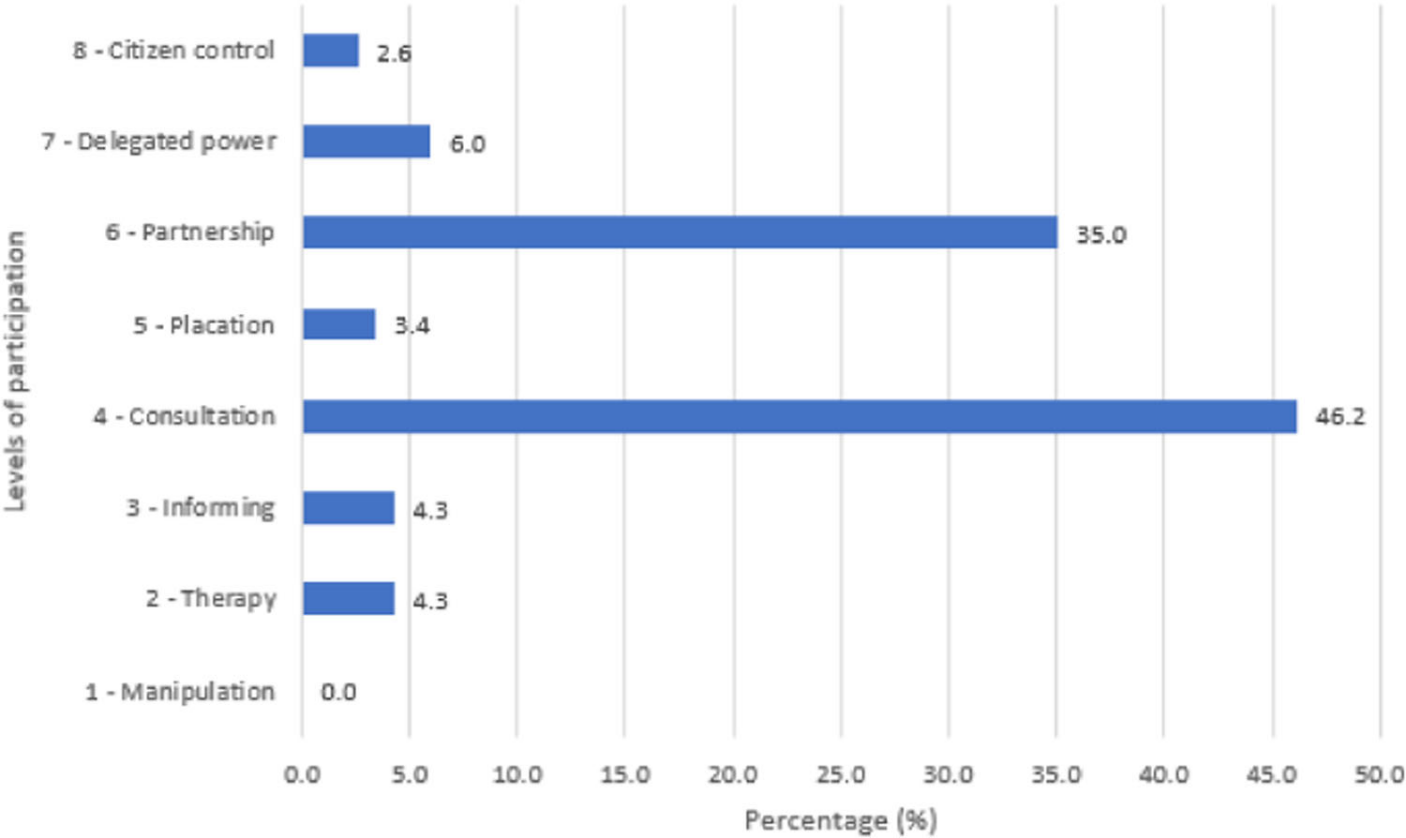
Level of stakeholders' participation during the identification and prioritization of research topics and subtopics (%), using Arnstein's levels.^16^

As Figure 4 shows, consultation (46.2%) and partnership (35%) were the most frequent levels of stakeholder participation in identifying and prioritizing the research (sub‐)topics. Although consultation is a low level of participation, the projects using it also applied methods with bidirectional exchange of ideas, such as workshops. A few projects (4.3%) only used therapy, and these were excluded from the analysis.

**Figure 5:**
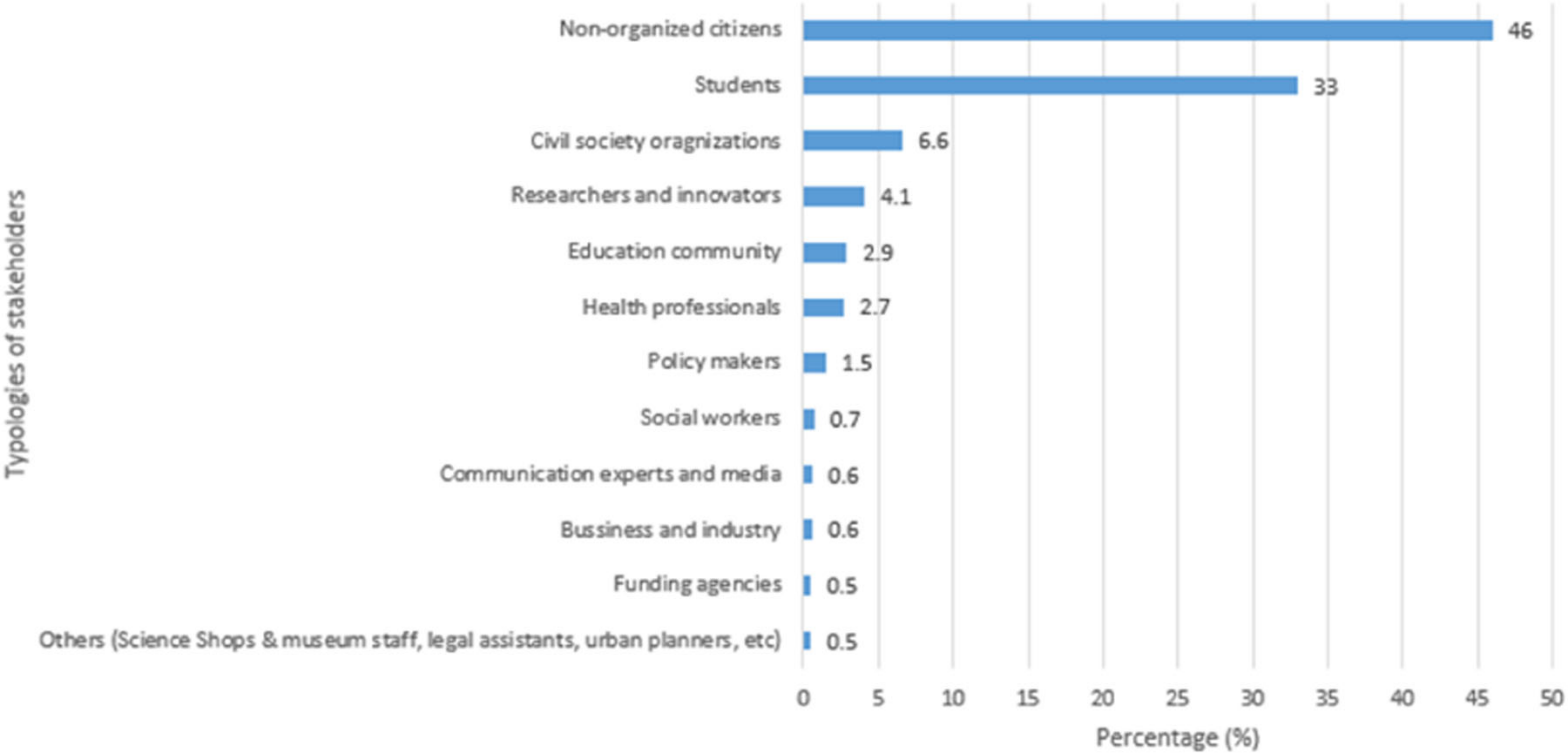
Percentage of typologies of stakeholders participating during the research agenda setting processes (%).

A wide diversity of stakeholders was involved in identifying and prioritizing research topics for the projects (see Figure 5). Of the 6035 participating stakeholders, the most common were nonorganized citizens (46%), who are citizens that do not participate as members or participants of an organization, and young people (students; 33%, as the Science Shop in Spain focused their projects on high schools).

### 3.2 Analysis of research fields

Here, we present the results of our analysis of research fields for the broad topic ‘Health’ and for the topics grouped in the 16 research fields. Some results in the following tables have been grouped into an ‘Others’ category because they were not relevant for our analysis. Thus, instead of 16 categories, the tables display 14 categories of research fields.

#### 3.2.1 Broad topic: Health

Table 4 shows percentages of WoS papers and Science Shop research projects, distributed by research fields. Within the broad topic ‘health’, WoS publications focused mostly on Clinical medicine (30.8%), followed by Health sciences (17.4%) and Biomedicine and molecular biosciences (16.7%). All other research fields have a much lower presence (5% or less). In contrast, Science Shops research projects mostly focused on Health sciences (56.2%) and the Social sciences (26%), with low involvement of Clinical medicine and Biomedicine and molecular biosciences (6.8% and 4.1%, respectively).

**Table 4:** Distribution amongst WoS publications and SS projects (%) across research fields at the global level.

	Health	
	WoS publications (%)	SS projects (%)
Agriculture, fisheries, forestry	3.8	0
Biology	1.6	2.7
Biomedicine and molecular biosciences	16.7	4.1
Business studies and economics	0.9	0
Chemistry	3.8	0
Clinical medicine	30.8	6.8
Engineering	4.3	0
Geosciences	3.2	0
Health sciences	17.4	56.2
Humanities	1	0
Physics	1.8	0
Psychology	2.8	4.1
Social sciences	5	26
Others	6.9	0
Total number	100	73

*Note*: The color scaling reflects the magnitude of the percentages, from red (low) to green (high).

Abbreviations: SS, Science Shop; WoS, Web of Science.

#### 3.2.2 Topics: HIV/AIDS, Chagas and Leprosy

Here we present our results regarding the percentages of WoS papers and Science Shop research projects on the topics of HIV/AIDS, Chagas and Leprosy.

Globally, WoS publications about *HIV/AIDS* were mostly focused on Clinical medicine (31.6%) and Biomedicine and molecular biosciences (25.6%), followed by Health sciences (16.5%) (Table 5). All other research fields were represented but with very low percentages. In contrast, most of the Science Shop research projects on HIV/AIDS focused on Health sciences (47%) and Social sciences (40%), with fewer focusing on Clinical medicine (13%) and none on Biomedicine and molecular biosciences. Country distribution of WoS publications in HIV/AIDS followed a similar pattern to the global distribution. In all countries, Clinical medicine was the main research field and Biomedicine and molecular biosciences were second (except in Ecuador, where Social Sciences was second). Low‐and middle‐income countries were substantially less present in Biomedical and molecular bioscience studies, as shown in comparing the countries outside Europe with The Netherlands and Spain (except for Bolivia, although it might not be relevant as results were based on only seven papers). Health sciences publications on HIV/AIDS differed somewhat between countries: from 7.4%, 8.8% and 12.6% in Tunisia, Spain and The Netherlands, respectively, to 17.5% and 23% in Ecuador and Nigeria. Again, the Social sciences were poorly represented. Psychology and Social sciences papers combined constituted 22.9% of the papers from Ecuador, but in other countries, the numbers are still very low: 4.9% in Spain, 5.3% in The Netherlands, 5.6% in Tunisia and 6.4% in Nigeria. By contrast, at the country level, Science Shop research projects were again mainly using Social sciences and Health sciences, except in Bolivia, where there were no papers in Social sciences, and in Ecuador, where all their projects were focused on Health sciences.

**Table 5:** Distribution of HIV/AIDS WoS publications and SS projects (%) across research fields, globally, and in selected countries.

	World		Spain		Bolivia		Ecuador		The Netherlands			Nigeria		Tunisia	
	WoS	SS	WoS	SS	WoS	SS	WoS	SS	WoS	SS partner	SS project	WoS	SS	WoS	SS
Agriculture, fisheries, forestry	1	0	1	0	0	0	0	0	0.6	0	0	0.8	0	3.7	0
Biology	2	0	1.4	0	11.1	0	7	0	1.4	0	0	0.9	0	1.9	0
Biomedicine and molecular biosciences	25.6	0	34.1	0	33.3	0	8.8	0	29.5	0	0	20.2	0	18.5	0
Business studies and economics	0.5	0	0.6	0	0	0	0	0	0.6	0	0	0.4	0	7.4	0
Chemistry	2.4	0	2.4	0	0	0	3.5	0	0.8	0	0	0.6	0	1.9	0
Clinical medicine	31.6	13.3	33.8	0	33.3	40	31.6	0	39.9	0	0	37.8	0	35.2	0
Engineering	2.4	0	1.7	0	0	0	0	0	1.2	0	0	0.7	0	3.7	0
Geosciences	1	0	1.4	0	11.1	0	3.5	0	0.9	0	0	0.4	0	0	0
Health sciences	16.5	46.7	8.8	33.3	0	60	17.5	100	12.6	50	0	23	100	7.4	50
Humanities	0.8	0	0.5	0	0	0	0	0	0.5	0	0	0.3	0	1.9	0
Physics	1	0	0.7	0	0	0	1.8	0	0.6	0	0	0.4	0	3.7	0
Psychology	3.3	0	1.8	0	0	0	1.8	0	2.8	0	0	2.1	0	1.9	0
Social sciences	4	40	3.1	66.7	11.1	0	21.1	0	2.5	50	100	4.3	0	3.7	50
Others	7.7	0	8.6	0	0	0	3.6	0	6	0	0	8.1	0	9.3	0
Unclassified	0.2	0	0.1	0	0	0	0	0	0.1	0	0	0	0	0	0
Total number of publications analyzed	63.021	15	2.340	6	7	5	50	1	1.844	2	1	915	1	41	2

*Note*: The color scaling reflects the magnitude of the percentages, from red (low) to green (high).

Abbreviations: AIDS, acquired immunodeficiency syndrome; HIV, human immunodeficiency virus; SS, Science Shop; WoS, Web of Science.

Both globally and in Spain (Table 6), WoS publications on Chagas were mostly biomedical (almost 50%), and about 27% of the papers were in Clinical medicine. In Bolivia, the publications were more evenly distributed amongst these two research fields (35.4% in each). In all cases, studies in the Social sciences and Health sciences were practically nonexistent. Amongst the Science Shop research projects, there were no biomedical projects at the global level nor in Spain and Bolivia, and Clinical medicine was a minor field (10% globally, 0% in Spain, 12.5% in Bolivia). Health sciences and Social sciences represented important research fields (40% globally, 50% in Spain, 37.5% in Bolivia).

**Table 6:** Distribution of Chagas WoS publications and SS research projects (%) across research fields, globally and in selected countries.

	World		Spain		Bolivia	
	WoS	SS	WoS	SS	WoS	SS
Agriculture, fisheries, forestry	4	0	2.7	0	2.5	0
Biology	4.7	0	1.3	0	3.8	0
Biomedicine and molecular biosciences	47.5	0	46.6	0	35.4	0
Business studies and economics	0	0	0	0	0	0
Chemistry	6.7	0	8.8	0	5.1	0
Clinical medicine	26.1	10	27.1	0	35.4	12.5
Engineering	0.5	0	0.6	0	1.3	0
Geosciences	0.5	0	0.4	0	0	0
Health sciences	4.8	50	6.5	50	6.3	50
Humanities	0.4	0	0	0	0	0
Physics	0.2	0	0.2	0	0	0
Psychology	0	0	0	0	0	0
Social sciences	0.4	40	0.4	50	0	37.5
Others	3.8	0	5.4	0	10.2	0
Total number	3610	10	327	2	48	8

*Note*: The color scaling reflects the magnitude of the percentages, from red (low) to green (high).

Abbreviations: SS, Science Shop; WoS, Web of Science.

Leprosy publications in WoS had many similarities with Chagas publications. As Table 7 shows, there was a lack of studies in Social sciences (no publications in the countries shown except The Netherlands (0.3%) and only 0.6% at the global level) and few studies in Health sciences at global level and in the selected countries (5.1% at the global level, and 4.6%, 9.6% and 8.2% in The Netherlands, Ethiopia and Nepal, respectively). However, there was an overwhelming dominance of Biomedicine and molecular biosciences (between 29.6% and 42.2% at the global level and in the countries shown) and Clinical medicine studies (between 34.7% and 49.3% at the global level and in the countries shown), and the percentage of Clinical medicine studies was much higher than it was for Chagas publications. Moreover, while The Netherlands had equal shares of Biomedical and Clinical studies (both 42%), papers at the global level were much more dominated by Biomedical fields, with a percentage of 41.1% (the same was found for countries such as the United States with 47.5%, not shown in the table). Furthermore, in Ethiopia and Nepal, the shares of Clinical studies were almost 45.6% and 49.3%, respectively, whereas publications from the Health sciences were relatively more frequent than in The Netherlands and at the global level (9.6% in Ethiopia and 8.2% in Nepal, compared to 4.6% in The Netherlands and 5.1% at the global level). In contrast, all the Science Shop research projects focused on Health sciences, with no representation from any other research field.

**Table 7:** Distribution of Leprosy WoS publications and SS projects (%) across research fields, at the global level and in selected countries.

	World		The Netherlands (partner country)		Ethiopia (project implementation)		Nepal (project implementation)	
	WoS	SS	WoS	SS	WoS	SS	WoS	SS
Agriculture, fisheries, forestry	2.8	0	0.9	0	1.6	0	0	0
Biology	1.2	0	0.9	0	0	0	0	0
Biomedicine and molecular biosciences	41.1	0	42.2	0	29.6	0	37	0
Business studies and economics	0	0	0	0	0	0	0	0
Chemistry	5.2	0	0.9	0	0	0	0	0
Clinical medicine	34.7	0	42.8	0	45.6	0	49.3	0
Engineering	1.1	0	0.6	0	0	0	0	0
Geosciences	0.8	0	1.1	0	0.8	0	0	0
Health sciences	5.1	100	4.6	100	9.6	100	8.2	100
Humanities	0.8	0	0	0	0	0	0	0
Physics	0.7	0	0.3	0	0	0	0	0
Psychology	0	0	0	0	0	0	0	0
Social sciences	0.6	0	0.3	0	0	0	0	0
Others	6	0	5.5	0	12.8	0	5.5	0
Total number	4.968	4	254	4	93	1	47	3

*Note*: The color scaling reflects the magnitude of the percentages, from red (low) to green (high).

Abbreviations: SS, Science Shop; WoS, Web of Science.

Comparisons between the countries reveal how Science Shops contributed to the development of research projects on specific diseases in low‐ and middle‐income countries where the total number of publications on those topics was low, but the national burden of disease was substantial (according to 2019 measures of disability‐adjusted life years (DALYs) from The Global Health Observatory^21^). That was the case for HIV/AIDS in Bolivia, with 7 publications out of 63,021 worldwide, and the DALYs represented 0.3% of the total disease burden in the country, and in Tunisia, with 41 publications and the DALYs represented 0.4% of the total disease burden in the country. For Chagas, there were 48 publications in Bolivia, out of 3610 worldwide, and the DALYs represented 0.6% of the country's total disease burden. For Leprosy, although the DALYs in Nepal and Ethiopia did not make up high percentages of these countries' national disease burdens (0.003% and 0.002%, respectively), these countries are amongst the 16 countries with the highest number of new cases of leprosy (2000+ each year). Just three countries report more than 10,000 new cases each year (India, Brazil and Indonesia). Despite the high numbers of new cases in these two countries, there were only 47 (Nepal) and 93 publications (Ethiopia) publications on leprosy, out of 4968 worldwide.

## 4 DISCUSSION

This study has shown that the Science Shop research projects mostly identified and prioritized research topics through participatory processes with a wide diversity of stakeholders, mainly nonorganized citizens and young people (high school students). Furthermore, the Science Shop projects made much more use of Social sciences and Health sciences, mainly focusing on nondrug treatments, compared to the global publications in WoS, which focused on Clinical medicine and Biomedicine and biomolecular sciences, largely aiming at the development of drug treatments. This difference in research focus is very much in line with findings from previous studies on research agenda bias.^10^, ^11^, ^12^ We, therefore, argue that the Science Shop projects contributed to decreasing existing bias in health research agenda setting, where research on nondrug treatments is underrepresented.

Comparing the three subtopics, Science Shops implemented research on diseases in countries where there was a substantial disease burden that was receiving little attention from the research community. This lack of attention had led to an agenda bias between treatments for illnesses affecting high‐income countries versus low‐ and middle‐income countries and to a lack of local solutions in those latter countries. Our research has shown that Science Shops' research contributed to decreasing this bias, for instance regarding HIV in Bolivia and Tunisia, and Chagas in Bolivia. For Leprosy, the Science Shops contributed to research in Nepal and Ethiopia, where the disease burden of this illness was not substantially high, but the numbers of new cases reported per year were amongst the highest in the world.

The WoS results (Tables 5, 6, 7) also indicate a global ‘division of labour’ in the distribution of research fields in publications. Low‐ and middle‐income countries are more involved in projects ‘on‐site’, while more basic research and development are carried out at laboratories in high‐income countries.^28^, ^29^ This finding is supported by, for example, publications on leprosy, which in Ethiopia and Nepal are mainly focused on Clinical medicine, whereas global and US publications are primarily focused on Biomedicine and molecular biosciences. HIV/AIDS publications show a similar pattern. Further investigations could explore if this pattern is also adopted by Science Shop research projects. The Science Shops all used participatory methods with a high degree of participation and a wide diversity of stakeholders to identify and prioritize their research focus, which is in line with the main aims and values of Science Shops and the new approaches to research agenda setting.^1^, ^2^, ^8^ However, methods that follow an iterative approach to identify research needs, such as the Dialogue Model or the Delphi method, were used less frequently (3.8% and 1.1% of the cases, respectively), while these methods have been shown to be more effective than the other ones, especially for complex problems.^9^ Further research is needed on what type of participatory methods are more effective for facilitating the implementation of the resulting agendas and decreasing the research agenda bias, and on the barriers to implementing these methods and the resulting agendas. Such research should pay specific attention to understanding how these barriers to implementation vary in different cultural contexts, as, for example, cultural norms on who should participate in agenda setting and the role of research may be very different in Africa, South America and in Europe. This research is key if the Science Shop model is to be scaled up to different cultural contexts.

### 4.1 Limitations

Our research assumed that (1)research publications in WoS are based on researchers' and funders' priorities and that (2) agenda bias can be redressed by participatory approaches, such as those implemented by Science Shops. However, the priorities might not be implemented due to different barriers, such as research group traditions and interests, academic power relations, career ambitions, societal lobbies, political and policy preferences, resources available, who sponsors the research (e.g., university, CSO, research funding organization), or the predominance of some professions in research (e.g., towards medical and surgical fields).^11^, ^12^ These various barriers can only be addressed with methods that follow an iterative approach, where the different stakeholders can reflect together in action‐learning spirals, and identify not only the research priorities but also the systemic barriers and possible strategies to address them.

Regarding the diversity of stakeholders involved, the Science Shops participating in the InSPIRES project are not representative of Science Shops in general. Science Shops usually follow the more traditional model in which CSOs are the main stakeholder group.^1^, ^2^, ^9^ The Science Shops in our study followed the open and inclusive approach of the latest Science Shop models. Involving a higher diversity of stakeholders in defining and prioritizing research projects may increase the likelihood that the research will be aligned with their needs and that systemic barriers will be addressed and thus that this approach will contribute (more) to decreasing existing bias. We therefore suggest that Science Shops evolve towards involving a larger diversity of stakeholders. In our study, the diversity of stakeholders could have been improved with a better representation of business/industry and funding agencies, which were hardly involved. Therefore, further research is needed on the diversity of stakeholders types required, and on similar comparisons regarding approaches for ‘recruiting, retaining, or ensuring the inclusion of diverse and/or representative stakeholders in governance activities’^18^ and also for addressing the systemic barriers.

Another limitation is the use of the Arnstein Ladder of Participation. The model has been criticized for: neglecting power relations and differences,^24^ ignoring different forms of knowledge and expertise,^30^ reducing participation to ‘a hierarchical set of social relations that are devoid of context’,^31^ considering ‘consultation’ as participatory process, even though it does not facilitate the necessary reflexive processes, and lacking ‘insights into how participation might be progressed’.^31^ Despite these shortcomings, we particularly used Arnstein's model to label the methods being used in Science Shops, including those that were nonparticipatory (which enabled us to exclude projects), and those that may not facilitate bidirectional reflexivity and shared activity but at least give voice to stakeholders who are not participating in most of the decision‐making regarding today's research agendas. It is important to note that, in all InSPIRES projects, we found that consultation methods were combined with other more participatory methods, such as workshops. Nevertheless, follow‐up research is needed to dive deeper into the participatory agenda‐setting processes used by Science Shop to gain in‐depth insight into the participatory process with respect to power relations, context, reflexivity and impact. Other models are more suitable to guide such a deep dive.^30^, ^31^

Finally, we analyzed Science Shop projects contributing to the agenda bias between drug and nondrug interventions and within and between countries with high and low GDP per capita. However, there may be other biases in research agendas that should be the focus of further research.^11^, ^12^

## 5 CONCLUSIONS

Our results have shown that the Science Shop projects included in this study identified, prioritized, and implemented research projects that contribute to decreasing the research agenda bias between: (1) drug and nondrug treatments and (2) between treatments in countries with high‐income and middle‐ and low‐income countries. Therefore, this study gives the first evidence of the effectiveness of Science Shops in their potential to address current health research agenda bias.

We conclude that there is a need for increased support for Science Shops or similar intermediary structures between researchers and other stakeholders to play a key role in contributing to shaping local, national, and international research policies. They should, however, be provided with sufficient resources to ensure that their research priorities address current research agenda biases by using participatory methods that involve a wide diversity of stakeholders and consider the stakeholders' needs while dealing with systemic barriers. For the most complex challenges, this will only be possible by applying methods for participatory research agenda‐setting that follow an iterative approach. Such methods allow different stakeholders to reflect together in action‐learning spirals and identify not only the research priorities but also systemic barriers and possible strategies to address them.

## AUTHOR CONTRIBUTIONS

**Aina Estany**: Data curation; visualization; validation; writing—original draft; formal analysis; investigation. **Fredrik Niclas Piro**: Formal analysis; validation; visualization; writing—original draft; investigation; writing—review and editing. **Jacqueline E. W. Broerse**: Writing—review and editing; validation. **Rosina Malagrida**: Validation; funding acquisition; conceptualization; writing—review and editing; methodology; writing—original draft; project administration; supervision; investigation; resources.

## CONFLICT OF INTEREST STATEMENT

The authors declare no conflict of interest.

## Supporting information

Supporting information.

## Data Availability

Data are available in the article Supporting Information S1: Appendices A and B.

## References

[1] Living Knowledge Network . *How Does a Science Shop Work*. Living Knowledge Network; 2023.

[2] Gnaiger A , Martin E . *Science Shops Operational Options*. SCIPAS Report No. 1. Living Knowledge; 2001.

[3] Fischer C , Leydesdorff L , Schophaus M . Science shops in Europe: the public as stakeholder. Sci Public Policy. 2004;31(3):199‐211.

[4] DeBok C , Steinhaus N . Breaking out of the local: international dimensions of science shops. Gateways Int J Community Res Engagement. 2008;1:165‐178.

[5] Mulder HAJ , Auf derHeyde T , Goffer R , Teodosiu C . *Success and Failure in Starting Science Shops*. SCIPAS Report No. 2; Living Knowledge; 2001.

[6] Kupper F , Klaassen P , Rijnen M , Vermeulen S , Broerse J . *Report on the Quality Criteria of Good Practice Standards in RRI*. RRI Tools. Athena Institute, VU University Amsterdam; 2015.

[7] Von Schomberg R . Prospectsfor Technology Assessment in a Framework of Responsible Research and Innovation. SSRN; 2014.

[8] Owen R , Macnaghten P , Stilgoe J . Responsible research and innovation: from science in society to science for society, with society. Sci Public Policy. 2012;39:751‐760.

[9] Urias E , Vogels F , Yalcin S , Malagrida R , Steinhaus N , Zweekhorst M . A framework for Science Shop processes: results of a modified Delphi study. Futures. 2020;123:102613.

[10] Crowe S , Fenton M , Hall M , Cowan K , Chalmers I . Patients', clinicians' and the research communities' priorities for treatment research: there is an important mismatch. Res Involv Engagem. 2015;1:2. 29062491 10.1186/s40900-015-0003-x PMC5598091

[11] Tallon D , Chard J , Dieppe P . Relation between agendas of the research community and the research consumer. Lancet. 2000;355:2037‐2040. 10885355 10.1016/S0140-6736(00)02351-5

[12] Knottnerus JA , Tugwell P . Research‐agenda bias. J Clin Epidemiol. 2018;98:vii‐viii. 29784131 10.1016/j.jclinepi.2018.04.020

[13] Balázs B , Gresle A‐S . *Report on the Potential Science Shop 2.0 Model. Transformative Ambitions, Impacts, Social Innovation Potentials*. InSPIRES; 2018.

[14] Abma TA , Broerse JEW . Patient participation as dialogue: setting research agendas. Health Expect. 2010;13:160‐173. 20536537 10.1111/j.1369-7625.2009.00549.x PMC5060528

[15] Muniz Pereira Urias E , Vogels F , Zweekhorst M . Results on the pilot projects on new models for Science Shops. 2019. Spring 2023. https://cordis.europa.eu/project/id/741677/results

[16] Malagrida R , Fernández J , Casabona J , Broerse JEW . A system‐oriented dialogue model to design community partnerships for more effective Sars‐Cov‐2 prevention in schools: the case of Spain. Int J Public Health. 2023;68:1605624. 37205045 10.3389/ijph.2023.1605624 PMC10186344

[17] Nygaard A , Halvorsrud L , Linnerud S , Grov EK , Bergland A . The James Lind Alliance process approach: scoping review. BMJ Open. 2019;9(8):e027473. 10.1136/bmjopen-2018-027473 PMC6720333 31473612

[18] Cope E , Dungan R , Isaacson A . *Strategies for Improving Patient Representativeness in Research Governance*. 2021 AcademyHealth.

[19] Kemmis S , McTaggart R . Participatory action research: communicative action and the public sphere. In: Denzin N , Lincoln Y , eds. Strategies of Qualitative Inquiry. Sage; 2008:271‐330.

[20] Balint PJ , Stewart RE , Desai A , Walters LC . Managing wicked environmental problems. Wicked Environmental Problems. Island Press; 2011:207‐217. https://link.springer.com/chapter/10.5822/978-1-61091-047-7_10

[21] Muniz Pereira Urias E , Zweekhorst M . Description of the new models for more inclusive Science Shops. 2019. Spring 2023. https://cordis.europa.eu/project/id/741677/results

[22] NordForsk . *Comparing Research at Nordic Higher Education Institutions using Bibliometric Indicators*. Policy Paper 4/2017. NordForsk; 2017.

[23] Arnstein SR . A Ladder of Citizen Participation. J Am Plann Assoc. 1969;35(4):216‐224.

[24] OpenLearn . Introducing the voluntary sector: week 6: 4.1 | OpenLearn—Open University. https://www.open.edu/openlearn/mod/oucontent/view.php?id=21024%A7ion=4.1

[25] Mongeon P , Paul‐Hus A . The journal coverage of Web of Science and Scopus: a comparative analysis. Scientometrics. 2016;106(1):213‐228.

[26] Petr M , Engels TCE , Kulczycki E , et al. Journal article publishing in the social sciences and humanities: a comparison of Web of Science coverage for five European countries. PLoS One. 2021;16(April):e0249879. 33831115 10.1371/journal.pone.0249879 PMC8031415

[27] Navas Fernández M. *Spanish Scientific Journals in Web of Science and Scopus Adoption of Open Access, Relationship Between Price and Impact, and Internationality*. TDX (Tesis Doctoralsen Xarxa). Universitat de Barcelona. 2016.

[28] Agnandji ST , Tsassa V , Conzelmann C , Köhler C , Ehni HJ . Patterns of biomedical science production in a Sub‐Saharan research center. BMC Med Ethics. 2012;13(1):3. 22448691 10.1186/1472-6939-13-3 PMC3382425

[29] Owusu‐Nimo F , Boshoff N . Research collaboration in Ghana: patterns, motives and roles. Scientometrics. 2017;110(3):1099‐1121.

[30] Tritter JQ , McCallum A . The snakes and ladders of user involvement: moving beyond Arnstein. Health Policy. 2006;76(2):156‐168. 16006004 10.1016/j.healthpol.2005.05.008

[31] Dedding C , Groot B , Slager M , Abma T . Building an alternative conceptualization of participation: from shared decision‐making to acting and work. Educ Action Res. 2023;31(5):868‐880. 10.1080/09650792.2022.2035788

