# Supplementary material for: Science Shops as key intermediary structures to respond to the current health research agenda bias: Evidence from the InSPIRES project
Source: Health Expect. 2024 Apr 23;27(2):e14052. doi: 10.1111/hex.14052 (PMC11039388; doi:10.1111/hex.14052)
Supplement: Supplementary file 1 — Supporting information. [file HEX-27-e14052-s001.docx]

**Appendix A**

**Table The NordForsk (2017) reclassification of Web of Science (WoS) subjects into 16 broad fields**

| **NordForsk field** | **Includes the following Web of Science categories** |
| --- | --- |
| **Agriculture, Fisheries & Forestry** | Agricultural Economics & Policy; Agriculture, Dairy & Animal Science; Agriculture, Multidisciplinary; Agronomy; Fisheries; Food Science & Technology; Forestry; Horticulture; Plant Sciences; Soil Science; Water Resources; Veterinary Sciences |
| **Biology** | Biodiversity Conservation; Biology; Ecology; Entomology; Evolutionary Biology; Limnology; Marine & Freshwater Biology; Mathematical & Computational Biology; Mycology; Ornithology; Zoology |
| **Biomedicine & Molecular Biosciences** | Anatomy & Morphology; Behavioural Sciences; Biochemical Research Methods; Biochemistry & Molecular Biology; Biophysics; Biotechnology & Applied Microbiology; Cell & Tissue engineering; Cell Biology; Chemistry, Medicinal; Developmental Biology; Genetics & Heredity; Immunology; Medical Laboratory Technology; Medicine, Research & Experimental; Microbiology; Microscopy; Neurosciences; Parasitology; Pathology; Pharmacology & Pharmacy; Physiology; Reproductive Biology; Toxicology; Virology |
| **Business Studies & Economics** | Business; Business, Finance; Economics; Management |
| **Chemistry** | Chemistry, Analytical; Chemistry, Applied; Chemistry, Inorganic & Nuclear; Chemistry, Multidisciplinary; Chemistry, Organic; Chemistry, Physical; Crystallography; Electrochemistry; Polymer Science |
| **Clinical Medicine** | Allergy; Andrology; Anaesthesiology; Audiology & Speech-Language Pathology; Cardiac & Cardiovascular Systems; Clinical Neurology; Critical Care Medicine; Dentistry, Oral Surgery & Medicine; Dermatology; Emergency Medicine; Endocrinology & Metabolism; Gastroenterology & Hepatology; Geriatrics & Gerontology; Haematology; Infectious Diseases; Medicine, General & Internal; Neuroimaging; Obstetrics & Gynaecology; Oncology; Ophthalmology; Orthopaedics; Otorhinolaryngology; Paediatrics; Peripheral Vascular Disease; Psychiatry; Radiology, Nuclear Medicine & Medical Imaging; Respiratory System; Rheumatology; Surgery; Transplantation; Tropical Medicine; Urology & Nephrology |
| **Computer & Information Sciences** | Computer Science, Artificial Intelligence; Computer Science, Cybernetics; Computer Science, Information Systems; Computer Science, Interdisciplinary Applications; Computer Science, Software Engineering; Computer Science, Theory & Methods |
| **Engineering** | Agricultural Engineering; Automation & Control Systems; Computer Science, Hardware & Architecture; Construction & Building Technology; Energy & Fuels; Engineering, Aerospace; Engineering, Biomedical; Engineering, Chemical; Engineering, Civil; Engineering, Electrical & Electronic; Engineering, Environmental; Engineering, Geological; Engineering, Industrial; Engineering, Manufacturing; Engineering, Marine; Engineering, Mechanical; Engineering, Multidisciplinary; Engineering, Ocean; Engineering, Petroleum; Green & Sustainable Science & Technology; Instruments & Instrumentation; Metallurgy & Metallurgical Engineering; Mining & Mineral Processing; Operations Research & Management Science; Robotics; Telecommunications; Thermodynamics; Transportation Science & Technology |
| **Geosciences** | Environmental Sciences; Geochemistry & Geophysics; Geography, Physical; Geology; Geosciences, Multidisciplinary; Meteorology & Atmospheric Sciences; Mineralogy; Oceanography; Palaeontology; Remote Sensing |
| **Health Sciences** | Gerontology; Health Care Sciences & Services; Health Policy & Services; Integrative & Complementary Medicine; Medical Ethics; Medical Informatics; Medicine, Legal; Nursing; Nutrition & Dietetics; Primary Health Care; Public, Environmental & Occupational Health; Rehabilitation; Social Sciences, Biomedical; Sport Sciences; Substance Abuse |
| **Humanities** | Archaeology; Architecture; Art; Asian Studies; Classics; Cultural Studies; Dance; Ethics; Film, Radio, Television; Folklore; History; History & Philosophy of Science; History of Social Sciences; Humanities, Multidisciplinary; Language & Linguistics; Linguistics; Literary Reviews; Literary Theory & Criticism; Literature; Literature, African, Australian, Canadian; Literature, American; Literature, British Isles; Literature, German, Dutch, Scandinavian; Literature, Romance; Literature, Slavic; Medieval & Renaissance Studies; Music; Philosophy; Poetry; Religion; Theatre |
| **Materials Science** | Materials Science, Biomaterials; Materials Science, Ceramics; Materials Science, Characterization & Testing; Materials Science, Coatings & Films; Materials Science, Composites; Materials Science, Multidisciplinary; Materials Science, Paper & Wood; Materials Science, Textiles; Nanoscience & Nanotechnology |
| **Mathematics & Statistics** | Logic; Mathematics; Mathematics, Applied; Mathematics, Interdisciplinary Applications; Statistics & Probability |
| **Physics** | Acoustics; Astronomy & Astrophysics; Imaging Science & Photographic Technology; Mechanics; Nuclear Science & Technology; Optics; Physics, Applied; Physics, Atomic, Molecular & Chemical; Physics, Condensed Matter; Physics, Fluids & Plasmas; Physics, Mathematical; Physics, Multidisciplinary; Physics, Nuclear; Physics, Particles & Fields; Quantum Science & Technology; Spectroscopy |
| **Psychology** | Psychology; Psychology, Applied; Psychology, Biological; Psychology, Clinical; Psychology, Developmental; Psychology, Educational; Psychology, Experimental; Psychology, Mathematical; Psychology, Multidisciplinary; Psychology, Psychoanalysis; Psychology, Social |
| **Social Sciences** | Anthropology; Area Studies; Communication; Criminology & Penology; Demography; Development Studies; Education & Educational Research; Education, Scientific Disciplines; Education, Special; Environmental Studies; Ergonomics; Ethnic Studies; Family Studies; Geography; Hospitality, Leisure, Sport & Tourism; Industrial Relations & Labour; Information Science & Library Science; International Relations; Law; Planning & Development; Political Science; Public Administration; Regional & Urban Planning; Social Issues; Social Sciences, Interdisciplinary; Social Sciences, Mathematical Methods; Social Work; Sociology; Transportation; Urban Studies; Women’s Studies |

**Appendix B: Dataset 3**

**Table Final selection of topics and projects for the analysis**

| **Broad Topic** | **Topic** | **Total no. of projects** | **Partner^[[1]](#footnote-1)^** | **Partner’s country** | **Country of implementation** | **Project** |
| --- | --- | --- | --- | --- | --- | --- |
| Health | HIV/AIDS | 10 | IrsiCaixa | Spain | Spain | Community-based participatory research project on HIV-related stigma in collaboration with secondary school students |
|  |  |  | IrsiCaixa | Spain | Spain | Access to the diagnosis of HIV among adolescents in Catalonia |
|  |  |  | IrsiCaixa | Spain | Spain | Co-creation of a social media communication campaign to prevent HIV and other STDs that is responsive to the needs identified previously |
|  |  |  | IrsiCaixa | Spain | Spain | Co-ResponsaHIVlity: Setting a R&I agenda on HIV and other STIs |
|  |  |  | CEADES | Bolivia | Bolivia | Evaluation of nutritional status through the Chang method applied in HIV-positive people undergoing ARV treatment in Cochabamba (Bolivia). |
|  |  |  | CEADES | Bolivia | Bolivia | Effect of HIV/AIDS diagnosis on risk behaviour for transmission of the disease in Cochabamba (Bolivia). |
|  |  |  | IPT | Tunisia | Tunisia | Understanding the perceptions and attitudes of health professionals, decision-makers, and civil society regarding medical care for PLHIV. It will help identify difficulties in patient pathways |
|  |  |  | VU | Netherlands | Netherlands | How the dialogue model can be used in Dutch HIV/AIDS and STI prevention |
|  |  |  | VU | Netherlands | Nigeria | Community responses regarding HIV: The game changer for equity and access to quality services for key and vulnerable populations in the context of universal health coverage in Nigeria |
|  |  |  | Organizations selected through an Open Call | Bolivia-Ecuador | Bolivia - Ecuador | Reduction of stigma and discrimination within the health system against people living with HIV/AIDS |
| Health | Chagas | 5 | ISGlobal | Spain | Spain | Access to healthcare for Chagas disease in Zaragoza, Spain |
|  |  |  | CEADES | Bolivia | Bolivia | Effectiveness of motivational training to improve vector control of Chagas in a rural community in Cochabamba (Bolivia) |
|  |  |  | CEADES | Bolivia | Bolivia | Study of the association of adverse effects and the restrictive diet during the treatment of Chagas disease |
|  |  |  | CEADES | Bolivia | Bolivia | Peer education as a model for communication in Chagas disease in Cochabamba (Bolivia) |
|  |  |  | CEADES | Bolivia | Bolivia | Living with Chagas disease: A qualitative study based on family stories in the Alto Valley of Cochabamba (Bolivia) |
| Health | Leprosy | 4 | VU | Netherlands | Nepal | Research priority setting of LRI (Leprosy Research Initiative). |
|  |  |  | VU | Netherlands | Nepal | Acceptability of leprosy post-exposure prophylaxis program in Nepal and actionable factors for future health policy uptake of post-exposure prophylaxis at the national level |
|  |  |  | VU | Netherlands | Ethiopia | Development of a protocol to determine the case detection delay of leprosy in the cultural context of East Hararghe Zone, Ethiopia: A cross-sectional mixed-methods cultural validation study |
|  |  |  | VU | Netherlands | Nepal | Cross-cultural validation of two scales to assess stigma and social participation of people with leprosy in Kathmandu Valley, Nepal |

1. IrsiCaixa- Institute for Aids Research, CEADES- Colectivo de Estudios Aplicados y Desarrollo Social Juan XXIII, IPT- Institute Pasteur de Tunis, VU- Vrije Universitait Amsterdam, ISGlobal-Instituto de Salud Global [↑](#footnote-ref-1)
